# Supplementary material for: Detection of SARS-CoV-2 from Saliva as Compared to Nasopharyngeal Swabs in Outpatients
Source: Viruses. 2020 Nov 17;12(11):1314. doi: 10.3390/v12111314 (PMC7697440; doi:10.3390/v12111314)
Supplement: Supplementary file 1 [file viruses-12-01314-s001.pdf]

SUPPLEMENTARY METHODS

Supplemental SARS-CoV-2 Testing of Positive Saliva Samples with Paired Negative NPS

All positive samples and a random subset of 23 negative samples were also tested using a second method different than Roche cobas SARS-CoV-2 assay used for primary characterization. This method involved initial offboard virus inactivation by combining 200 uL of each patient saliva sample with 250 uL of lysis buffer master mix, containing TNA lysis buffer (Omega Bio-tek, Inc., Norcoss, GA USA), Carrier RNA (Omega Bio-tek) and MS2 phage internal control (Thermo Fisher, Waltham, MA USA). RNA was extracted using MagBind Viral RNA Xpress kit (Omega Bio-tek) on Hamilton Microlab STARlets (Hamilton Company Reno, NV USA). A 10 uL aliquot of RNA was added in a 15 uL reaction using TaqPath COVID-19 Combo Kit (Thermo Fisher Scientific) on real-time PCR systems (Applied Biosystems 7500 Fast or QuantStudio 6); one cycle at 25°C for two minutes, one cycle at 53°C for 10 minutes, one cycle at 95°C for two minutes, 40 cycles at 95°C for three seconds and 60°C for 30 seconds. A sample was defined as positive if the viral genome was detected at threshold cycle (Ct) values of <37 and as negative at Ct values ≥37.

Saliva Self-Collection – Instructions to Participants

1. Wash and dry hands or apply an alcohol-based hand rub before opening the kit provided.
2. Open the kit and place all the contents on a clean, dry surface (collection tube, label, biohazard bag, moist towelette).
3. Affix label provided to the collection tube.
4. Screw off the top of the sterile collection tube.
5. Allow saliva to accumulate in the floor of the mouth for at least 60 seconds and then express the saliva into the collection container provided. Avoid contaminating the outside of the container with saliva.
6. Repeat saliva expression until at least 3 mL has been obtained as per the tube markings. This usually involves "spitting" 5-6 times into the tube.
7. Screw the top of the collection tube back on.
8. Wash and dry hands thoroughly again or apply an alcohol-based hand rub.
